# The impact of blood flow restriction training on tendon adaptation and tendon rehabilitation – a scoping review

**DOI:** 10.1186/s12891-025-08734-5

**Published:** 2025-05-22

**Authors:** Samuel Öberg, Ludvig von Schewelov, Eva Tengman

**Affiliations:** 1 Dept. of Community Medicine and Rehabilitation, Section for Physiotherapy, Umeå University, Umeå, SE-90187 Sweden; 2 Umeå School of Sport Sciences, Umeå University, Umeå, Sweden

**Keywords:** Tendinopathy, Tendon rupture, Rehabilitation, Tendon adaptation

## Abstract

**Background:**

Tendon injuries are common in athletes and in the general population and require extensive rehabilitation. Current conservative treatment often includes different high-load resistance training (HLRT) modalities. However, certain populations may not tolerate HLRT well. Blood flow restriction training (BFRT) incorporates low load while achieving hypertrophy and strength adaptations comparable to HLRT. However, the effects of BFRT on healthy and pathological tendons are unknown. The aims of this scoping review were therefore to summarize the reported impact of BRFT: (1) on tendon adaptation in healthy individuals, and (2) in tendon rehabilitation after injury.

**Methods:**

A scoping review based on PRISMA guidelines was performed. A systematic literature search in the electronic databases PubMed, SPORTDiscus and CINAHL was performed in May 2024. This review includes peer-reviewed articles investigating the effects of BFRT on healthy tendons and in tendon rehabilitation. Methodological quality was assessed using the Downs and Black scale and JBI Critical Appraisal Checklist.

**Results:**

19 studies with varied design, population, investigated tendon, intervention design and outcome measures were eligible. Ten studies were randomized controlled trials (RCT), one study was a clinical controlled trial, three studies were feasibility studies and five were case reports. The reviewed studies included 351 healthy subjects and 122 individuals with tendon-related disorders (101 subjects with tendinopathy and 21 subjects with tendon ruptures). Tendons investigated were Achilles (*n* = 6), patellar (*n* = 6), hamstring (*n* = 1), gluteal (*n* = 1), biceps brachii distal (*n* = 1), tendons of the rotator cuff (*n* = 2) and lateral elbow extensors (*n* = 2). In the nine studies on healthy individuals, the effects of BFRT showed contradictory results regarding tendon-related outcomes. However, changes in outcome measures did not differ significantly from HLRT conditions or low-load resistance training (LLRT) conditions. The studies on tendon rehabilitation also showed contradictory results regarding tendon-related outcomes, although several studies do report decreased pain, increased strength, enhanced performance and improved self-reported diagnosis-specific function.

**Conclusions:**

The present scoping review shows contradictory results regarding tendon-related outcomes although studies point to increasing tendon function after rehabilitation. BFRT may be a viable option to incorporate into training regimes aimed at inducing tendon adaptation. Further in-depth research is warranted.

**Clinical trial number:**

This is a review and therefore is Clinical trial number: Not applicable. However, the review has been preregistered at www.osf.io (DOI 10.17605/OSF.IO/PYV43) stated in the method section.

**Supplementary Information:**

The online version contains supplementary material available at 10.1186/s12891-025-08734-5.

## Background

Tendons have an essential role in the musculoskeletal system due to their capacity to store energy and transmit forces from muscle to bone. Like soft tissues, tendons adapt to loading or disuse through morphological and mechanical changes. Maintaining adequate tendon function is important for both athletic performance and activities of daily living [1]. Injuries to tendons often follow excessive or inadequate mechanical loading, which diminishes the tendon’s ability to maintain homeostasis and thus decreases function [2]. Two common tendon injuries are tendon ruptures and tendinopathy. Tendon ruptures and tendinopathy are disabling injuries and are common in both the upper and lower extremities among athletes and in the general population [3–7]. The most extensively researched tendon pathology is Achilles tendinopathy, which has an incidence of 2–3 per 1000 patients in general medical practice [3]. For the upper extremity, rotator cuff tendon tears are commonly seen in the general population. Incidence increases with age and 23–49% of individuals aged between 40 and 70 years have had partial- or full-thickness tears [8].

Exercise therapy is commonly used as an initial treatment for tendinopathy [9] and is an important part of tendon rupture rehabilitation [10, 11]. Previous research on Achilles and patellar tendinopathy using eccentric loading has led clinicians to recommend eccentric training as a primary treatment [12–14]. More recent reviews, however, have questioned the need to isolate the eccentric component, demonstrating similar results for dynamic concentric-eccentric training [15, 16]. Different high-loading modalities have shown to be beneficial. However, some populations respond to a lesser degree to high loading [15, 16]. Blood flow restriction training (BFRT) has shown promising results in the rehabilitation of painful musculoskeletal ailments such as knee osteoarthritis and anterior cruciate ligament injury, where BFRT has been shown to be useful when strength training exacerbates knee pain [17–19]. In addition, a systematic review with meta-analysis proposed the incorporation of BFRT as a tool in the rehabilitation of painful musculoskeletal conditions [20].

BFRT is commonly utilized with a torniquet applied proximally on the leg or arm to primarily restrict venous-, and secondly arterial blood flow to maximize metabolite accumulation in the working muscles [21]. The method utilizes low loads, 20–40% of repetition maximum (1RM), 15–30 repetitions and short rest periods [20]. In comparison to conventional HLRT (with > 70% 1RM), BFRT has been shown to produce similar adaptations in muscle mass [22, 23]. Although strength adaptations do not occur at the same rate as in HLRT, adaptations seem to be greater than in low-load resistance training (LLRT) [24, 25]. The mechanisms behind BFRT are not fully elucidated but it is hypothesized that the ischaemic condition causes metabolic stress, which enhances the effect of the mechanical tension applied [26]. Other factors contributing to strength and hypertrophy increases might be cell swelling, increases in type 2 fibre recruitment, hormone production and anabolic signalling within the muscle [24, 26]. How such physiological mechanisms contribute to morphological and mechanical tendon adaptations and its potential benefits in tendon rehabilitation needs to be further investigated. Smaller reviews, recently published, highlight the potential beneficial effects of BFRT in healthy and pathological tendons [27, 28]. Several trials examining the effects of BFRT in both healthy and pathological tendons have recently been published, which has been included in this scoping review contributing to a wider knowledge base [29–36]. An updated summary of the current evidence is therefore needed. The aims of this scoping review were to summarize the reported impact of BRFT: (1) on tendon adaptation in healthy individuals, and (2) in tendon rehabilitation after injury. The scoping review synthesizes study design, tendon, participant characteristics, sample size, intervention, measurements, outcome and conclusions.

## Methods

### Study design

A scoping review design was used in the present study. Scoping review has advantages when articles are scarce and heterogenic [37]. Randomized controlled trials, non-randomized trials and case reports were included in this review and the totality of eligible literature was scrutinized thoroughly. The PRISMA guidelines for scoping reviews (PRISMA-ScR) were used for compilation [37] and the review was preregistered at www.osf.io (DOI 10.17605/OSF.IO/PYV43).

### Search strategy

A systematic article search was performed in PubMed, CINAHL, and SPORTDiscus in May 2024. An experienced medical librarian assisted in developing the search string. Controlled vocabulary was used in the databases to construct an appropriate search string for each database (Supplementary material). Keywords included terminology associated with tendons, tendinopathy, and blood flow restriction training. Searches in PubMed and CINAHL were filtered by abstract. In addition to database searching, earlier reviews and the reference lists of included articles were screened for additional eligible papers.

### Eligibility criteria

Criteria for inclusion were primary research studies investigating the effects of BRFT on material- or tendon-morphological properties in healthy individuals or BRFT used for rehabilitation in patients with tendon ruptures or tendinopathy. Exclusion criteria were: studies not peer-reviewed, animal studies, and studies published in a language other than English or Swedish. The inclusion criteria regarding title and abstract screening were compiled to include broad enough terms to obtain relevant articles. All abstracts were independently screened according to the inclusion/exclusion criteria by two of the authors (ET, SÖ). Studies meeting the inclusion criteria proceeded to full-text screening (ET, SÖ), where outcome measures were scrutinized for eligibility.

### Data extraction

Two of the authors (LvS, ET) extracted data from the final set of articles. Data were categorized into author, study design, sample size, tendon, participant characteristics, intervention, and control conditions in Table 1, and measurements, outcome and conclusion in Table 2. During the critical appraisal, another author (SÖ) scrutinized extracted data for extraction errors.

**Table 1:** Overview of characteristics and interventions

First Author	Study Design & Sample size (*n*)	Tendon, sex & age	Intervention and Control condition
**Healthy individuals**			
Brumitt et al. 2020 [43]	Randomized controlled trial *n* = 46 (BFRT *n* = 24 LLRT *n* = 22)	Rotator cuff Healthy subjects 26 males 20 females Mean age 25 yrs	8 weeks of **BFRT** 2x/week, 4 sets of side lying external rotation, 30/15/15/15 repetitions. Cuff applied to the proximal portion of upper extremities 50%. Resistance set to 30%/1RM. **LLRT** Excluding the BFR-cuff, participants were given the same exercise and terms as the BFR-group.
Centner 2019 et al. [42]	Randomized controlled trial *n* = 37 (BFRT *n* = 11, HLRT *n* = 14 & CON *n* = 13)	Achilles tendon Healthy subjects Males 18-40 yrs	14 weeks of **BFRT** 3x/week, 4 sets of sitting and standing calf rise, 30/15/15/15 repetitions. Cuff applied in the proximal region of the thigh. Load starts at 20%/1RM, with 5% increase every fourth week. **CON** - No training intervention. 14 weeks of **HLRT** 3x/week, 3 sets of the same exercises as BFR, 6–12 reps. Resistance between 70–85%/1RM, progressed every fourth week.
Centner et al. 2022 [45]	Randomized controlled trial *n* = 29 (BFRT *n* = 14, HLRT *n* = 15)	Patellar tendon Healthy subjects Males 18-40 yrs Mean age 28 yrs	14 weeks of **BFRT** 3x/week, 4 sets of leg press, knee extension, sitting and standing calf rise, 30/15/15/15 repetitions. Cuff applied in the proximal region of the thigh, 50%. Load starts at 20%/1RM, with 5% increase every fourth week. **HLRT** 3x/week in 14 weeks, 3 sets of the same exercises as BFRT, 6–12 reps. Load starts at 70% progressively increased to 85%/1RM-.
Centner et al. 2023 [32]	Randomized controlled trial *n* = 29 (BFRT *n* = 14, HLRT *n* = 15)	Achilles tendon Healthy subjects Males 18-40 yrs Mean age 28 yrs	14 weeks of **BFRT** 3x/week, 4 sets of sitting and standing calf rise, 30/15/15/15 repetitions. Cuff applied in the proximal region of the thigh, 50%. Load starts at 20%/1RM, with 5% increase every fourth week. **HLRT** 3x/week in 14 weeks, 3 sets of the same exercises as BFRT, 6–12 reps. Load starts at 70% progressively increased to 85%/1RM-.
Chulvi-Medrano et al. 2020 [46]	Pilot clinical trial Intra-subject study design *n* = 56	Achilles tendon Healthy subjects 28 males 28 females Mean age 25 yrs	1 bout of **BFRT**, 3 sets of unilateral plantar-flexion assigned to dominant leg, 15/15/15 repetitions. Cuff applied below popliteal fossa, 30%. Load set to 30%/1RM. **LLRT** Excluding BFR-cuff, participants were given the same exercise and terms as the BFR-group. Intervention was applied to non-dominant leg.
Cintineo et al. 2024 [31]	Randomized controlled trial *n* = 54 (TRAD = 18 BFRT = 18 MIN = 18	Quadriceps Healthy subjects 32 males 22 females Mean age 21 yrs	Militaries performed whole body training (here focus results of effects on quadriceps). Cuff applied proximal with an increase the first 4 weeks then a decrease of occlusion, pressure percentage not stated. 6 weeks, 4x/weeks alternating 2 different workouts (9 + 9 exercises). Exercises for example different deadlifts, squats, calf rise, leg curl. **BRFT** 3 sets, 30-15-15 repetitions, **MIN** 3 sets, 30-15-15 repetitions, TRAD 3 sets, 3–10 repetitions.
Frouin et al. 2024 [33]	Randomized controlled trial *n* = 36 (BFRT = 13 HLRT = 11 CON = 12	Hamstrings Healthy subjects 23 males 13 females Mean age 22 yrs	9 weeks of **BFRT** 3x/week, 3 sets at 30%1RM with week 1–3 almost maximum and week 4–9 maximum repetitions. 4 exercises were alternated: stiff-leg deadlift, front squat, seated leg curl, seated leg extension with 2 exercises at each time. Cuff applied proximal thigh, 80%. **HLRT** performed same exercises. Week 1–3 3sets with 12 repetitions. Then increased up to 5 sets with maximum repetitions. **CON** continued their regular sport activities.
Kubo et al. 2006 [41]	Randomized controlled trial *n* = 9	Patella tendon Healthy subjects 9 Males 23–27 yrs	12 weeks of **BFRT** 3x/week, 4 sets of unilateral knee extension, 15/18/15/12 repetitions. Cuff applied proximally with an increase in occlusion pressure every fourth week, pressure percentage not stated. Resistance set to 20%/1RM. 12 weeks of **HLRT** 3x/week, 4 sets of sitting knee extensions, 10,10,10,10 repetitions. Load starts at 80%/1RM, weight adjusted in accordance with 80%/1RM every fourth week.
Picón-Martínez et al. 2021 [44]	Randomized controlled trial *n* = 54 (BFRT = 24, HLRT = 15, LLRT = 13)	Achilles Tendon Healthy subjects 33 males 19 females Mean age 27 yrs	4 sets of straight leg plantar flexion. **BFRT** 30/15/15/15 repetitions at 30%1RM with cuff applied below the popliteal fossa, 30%, **HLRT** 30 repetitions at 30%1RM and 3 × 10 at 75% 1RM. **LLRT**-group were given the same conditions as BFRT-group excluding the cuff.
**Individuals with tendon related disorders**			
Bentzen et al. 2024 [30]	Feasibility study, Intra-subject study design *n* = 18	Achilles tendon rupture 14 males 4 females Mean age 52 yrs	Initially treated with a cast. 12 weeks of **BFRT** 3x/week. Cuff applied proximal thigh. Phase (1) 4 sets seated knee extension 40% occlusion, 30/15/15/>15 repetitions, Phase (2) 4 sets seated knee extension 80% occlusion, 30/15/15/>15 repetitions. Phase (3) 4 sets seated knee extension 80% occlusion, 30/15/15/>15 repetitions and 5 × 2 min BFR walking.
Cuddeford et al. 2020 [48]	Case report 2 decathletes	Patellar tendinopathy Male 19 yrs	20 therapy-sessions of **BFRT** performed over 12 weeks, 4 sets of unilateral leg-press, 30,15,15,15 repetitions. 4 sets of unilateral squat on 25° squat board (initiated 3rd week), 30,15,15,15 (week 3–4) and adjusted to 15-15-15 (from 5:th week) repetitions. Cuff applied proximal to the lower limb, 80%. Load starts at 30%/1RM and participants were encouraged to add 10 lbs session.
Hogsholt et al. 2022 [34]	Feasibility study, Intra-subject study design *n* = 16	Gluteal tendinopathy 16 females Median age 51 yrs	8 weeks 7x/week, Week 1–2; 5 × 5s static standing abduction, 10 side step, 10 glute bridging, 10 squats *withou*t BRFT. Week 4–8 Daily static abduction and side stepping *without* BRFT and every second day glute bridging and squat *with* BRFT cuff application proximal thigh with 60% started with 2 set up to 3 sets week 7–8.
Kara et al. 2024 [29]	Randomized controlled trial *n* = 28 (BFRT = 14, LLRT = 14	Rotator cuff tendinopathy 14 males 14 females Mean age 31 and 35	8 weeks of **BFRT** 2x/week, 4 sets of 30/15/15/15 repetitions at 30%1RM. 4 exercises to activate rotator cuff muscles, biceps brachii, scapular stabilizer. Cuff applied to the proximal portion of the painful shoulder at 50%. **LLRT** performed same exercise protocol without the cuff
Karanasios et al. 2022 [36]	Randomized controlled trial *n* = 46 (BFRT = 23, LLRT sham = 23	Lateral elbow tendinopathy 23 males 22 females Mean age 45 yrs	6 weeks of **BFRT** 2x/week, 4 sets of 30/15/15/15 repetitions at 30%1RM of elbow flexion and extension. Cuff applied proximal, 40–50%. Then wrist flexion, extension, supination and pronation 3sets of 10 repetitions with a load with a pain of maximum VAS 2. After about 2weeks a addition of wall push-ups, wrist ext-flex, hand grip, rowing. **LLRT-sham** performed same exercise protocol with a “sham” cuff.
Karanasios et al. 2023 [35]	Case report 1 recreational tennis player	Lateral elbow tendinopathy Male 51 yrs	6 weeks of **BFRT** 2x/week, 4 sets of 30/15/15/15 repetitions at 30%1RM of wrist extension, Cuff applied proximal 40%0.3 sets of 10 repetition of wrist flexion, supination-pronation, elbow flexion and extension. Then wrist flexion, extension, supination and pronation 3sets of 10 repetitions with a load with a pain of maximum VAS 2. After about 2weeks a addition of wall push-ups, wrist ext-flex, hand grip, rowing.
Sata 2005 [51]	Case report 1 basketball player	Patellar tendinopathy 1 male 17 yrs	3 weeks of **BFRT 5-6**x/week, 3 sets of 15 repetitions at 30%1RM. SLR, hip add/abduction, calf raise, squat, shooting. Cuff applied proximal leg with 160–180 mmHg, percent not stated. The patient was also treated with anti-inflammatory drugs.
Skovlund 2020 [47]	Feasibility study, Intra-subject study design *n* = 7	Patellar tendinopathy 7 males Mean 29 yrs	3 weeks of **BFRT** 3x/week. 6 sets with 30/25/20/15/10/5 of unilateral leg press and knee extension. Load set to 30%/1RM. Cuff applied proximal with pressure of 120 mmHg, percent not stated..
Wentzell 2018 [50]	Case report 1 weightlifter	Distal biceps brachii rupture 1 male 35 yrs	12 weeks of **BFRT** 2x/week, 4 sets of elbow flexion, 30/15/15/15 repetitions. Cuff pressure of 80 mm/hg Week 1 and 2 without applied resistance + wrist at neutral position. week 3 resistance applied with 1 lbs Week 4 & 5, 1 lbs, week 6 & 7 resistance increased to 1,5 lbs and week 10–14 resistance increased to 4 lbs. Additional exercise* and passive treatments** applied during intervention. BFRT initiated in week 3 of intervention.
Yow et al. 2018 [49]	Case report 2 soldiers	Achilles tendon rupture 2 males 29-38 yrs	**BFRT** consisting of 4 sets of calf press and leg press, 30,15,15,15 repetitions. Cuff applied proximally to the thigh 80%. Load set to 30%/1RM. **S1** experiencing tendon rupture and enrolling in 5 weeks of BFRT 6 months post injury. **S2** experiencing contralateral tendon rupture and enrolling in 6 weeks of BFRT 4 months post injury.

**BFRT =** Blood flow restriction training **LLRT =** Low load-resistance training **LIRT =** Low-intensity resistance training **HLRT =** High load-resistance training **CON =** Control group **S1 =** Subject 1 **S2 =** Subject 2 **1RM** = 1 repetition maximum **yrs =** years

*Additional exercises = Open kinetic chain controlled articular rotations; Passive and active movements of the elbow and wrist Isometric of the elbow and wrist; Dynamic strength of upper body. All exercises increased resistance during intervention

**Passive treatments = Soft tissue therapy, Lymphatic drainage, Kinesiotape application, Laser therapy and Scar mobilization techniques

**Table 2:** Overview of outcomes, measurements and conclusions

First Author	Measurement	Outcome	Conclusion	Critical Appraisal
**Healthy individuals**				
Brumitt et al. 2020 [43]	1RM dynamometer - Shoulder abduction	**BFRT** ↑ 21.51% **LLRT** ↑ 22.25% (P = 0.75)	BFRT and LLRT increased supraspinatus tendon thickness and rotator cuff strength. The addition of BFRT did not result in greater increases compared to LLRT alone.	**D&B** 14/17
	1RM dynamometer - Shoulder external rotation	**BFRT** ↑ 49.2% **LLRT** ↑ 48.84% (P = 0.7)		
	U/S d - Supraspinatus tendon thickness	**BFRT** ↑ 3.98% **LLRT** ↑ 3.9% (P = 0.6)		
		P values are differences between groups		
Centner et al. 2019 [42]	U/S - CSA Achilles tendon Youngs modulus - Achilles tendon stiffness Maximum voluntary contraction U/S - CSA Gastrocnemius	**BFRT** ↑ 7.77% **HLRT** ↑ 4.55% **CON** ↑ 0.15% **BFRT** ↑ 36.15% **HLRT** ↑ 40.67% **CON** ↑ 3.62% **BFRT** ↑ 9.79% **HLRT** ↑ 13.54% **CON** ↓ 1.44% **BFRT** ↑ 9.09% **HLRT** ↑ 7.69% **CON** ↑ 0.7% All values are P < 0.05	BFRT and HLRT induce similar adaptations in the Achilles tendon. Additionally hypertrophic adaptations were comparable, and both groups experienced significant strength gains in the plantar flexors.	**D&B** 15/17
Centner et al. 2022 [45]	1RM leg press MRI – CSA rectus femoris muscle MRI – CSA patellar tendon U/S patellar tendon stiffness	**BFRT** ↑ 34% **HLRT** ↑ 37% **BFRT** ↑ 3% **HLRT** ↑ 6% **BFRT** ↑ 9% **HLRT** ↑ 4% **BFRT** ↑ 25% **HLRT** ↑ 22% All values pre-post was P < 0.05. no sign group effect except for CSA patellar tendon	The result show substantial changes in patellar tendon properties. and the magnitude of changes is not significantly different between conditions	**D&B** 13/17
Centner et al. 2023 [32]	MRI- CSA achilles tendon 1RM plantar flexion strength	**BFRT** ↑ 5.2% **HLRT** ↑ 5.3% **BFRT** ↑ 44% **HLRT** ↑ 43% All values pre-post was P < 0.05. no sign group effect.	The finding demonstrate similar pattern in Achilles tendon hypertrophy and increased plantar flexor strength in both groups.	**D&B** 13/17
Chulvi-Medrano et al. 2020 [46]	U/S - Acute Achilles tendon thickness U/S - Post 24h Achilles tendon thickness	**BFRT** ↓ 14.5% **LLRT** ↓ 9.67% **BFRT** ↓ 9.2% **LLRT** ↓ 1.06% All Values are P ≤ 0.05	Acute and subacute Achilles tendon thickness response to exercise differed between BFRT and LLRT groups. BFRT demonstrated greater decrease in tendon thickness and slower recovery than LLRT.	**D&B** 9/17
Cintineo et al. 2024 [31]	U/S – Rectus femoris/Quadriceps thickness U/S – Quadriceps tendon thickness	↑ Time main effect (p < 0.001), no group effects No differences (p = 0.225)	No significant differences were observed in quadriceps tendon thickness either for group or time. Strength improved, with greater improvements in TRAD group.	**D&B** 13/17
Frouin et al. 2024 [33]	U/S – semitendinosus tendon volume	No significant effect	.None of the training regimes induced significant hypertrophy of the semitendinosus tendon. BRFT induced hypertrophy of semimembranosus, while HLRT induced hypertrophy of the semitendinosus tendon.	**D&B** 13/17
	U/S –muscle volume – biceps long head	**BFRT** ↑ 14% **HLRT** ↑ 4% **CON** ↑ 1% NS		
	U/S –muscle volume –semitendinosus	**BFRT** ↑ 11% **HLRT** ↑ 27% **CON** ↓ 0.1% (HLRT- CON p = 0.004)		
	U/S –muscle volume -semimembranosus	**BFRT** ↑ 22% **HLRT** ↑ 17% **CON** ↑ 0.3% (BFRT-CON p = 0.025)		
	Isometric knee flexion torque Nm/kg	**BFRT** ↑6.2% **HLRT** ↑9.5% **CON** ↓0.3% (HLRT-CON p = 0.004)		
Kubo et al. 2006 [41]	U/S - Tendon-aponeurosis complex stiffness	**BFRT** ↑ 9.31% **HLRT** ↑ 30.04%	HLRT increased patella tendon stiffness and tendon-aponeurosis stiffness, while BFRT did not. No increase in patellar tendon CSA was observed. Both groups experienced significant strength increases in the knee extensors and quadriceps CSA.	**D&B** 12/17
	U/S - Patella tendon stiffness (MVC)	**BFRT** ↑ 1.65% **HLRT** ↑ 8.53%		
	U/S - CSA of patella tendon	**BFRT** ↓ 0.63% **HLRT** ↓ 0.64%		
	Dynamometer - MVC knee extension	**BFRT** ↑ 9.3% **HLRT** ↑ 14.2%		
	MRI - CSA Quadriceps	**BFRT** ↑ 7.4% **HLRT** ↑ 7.08%		
		All values are P < 0.05 except quadriceps volume (P = 0.195) and patella tendon CSA.		
Picón-Martínez et al. 2021 [44]	U/S - Acute Achilles tendon thickness U/S - Achilles tendon thickness post 60 min U/S - Achilles tendon thickness post 24h	**BFRT** ↓13.64% **HLRT** 0% **LLRT** ↑ 2.5% **BFRT** ↓15.91% **HLRT**↓2.22% **LLRT** 0% **BFRT** ↓6.82% **HLRT**↓2.22% **LLRT** ↑ 5%	Changes in Achilles tendon thickness acute, 60 min post and 24h post differed between groups. BFRT conditions showed a significant decrease in tendon thickness at all measurement points, whereas HLRT and LLRT conditions demonstrated significant changes tendon thickness.	**D&B** 13/17
**Individuals with tendon related disorders**				
Bentzen et al. 2024 [30]	Dropout rate Adherence and acceptability Adverse events. Ankle pain (NRS) Circumference of thigh and calf Achilles total rupture score Single-leg heel-rise	2 dropouts 88% and 92% 3 adverse events– 2 re-rupture and 1 DVT Mean NRS 1. 98% reported NRS ≤ 5 No diff between injured and non-injured leg ↓55% 13% were able to perform	BRFT was found to be feasible in terms of adherence, intervention acceptability, drop-out rate and pain. However, three adverse events occurred.	**JBI** 7/8 total yes
Cuddeford et al. 2020 [48]	U/S - Patella tendon thickness VAS Pain - During rest VAS Pain - During loading 1RM - Unilateral leg press Unilateral leg hop left leg VISA-P Questionnaire	**S1** ↓ 28.39% **S2** ↑ 6.98%* **S1** 2/10 − 0/10 **S2** 2/10 − 0/10 **S1** 8/10 − 0/10 **S2** 8/10 − 3/10 **S1** ↑ 60.58% **S2** ↑ 33.9% **S1** ↑ 3.03% **S2** ↑ 56.03% **S1** 45–82 **S2** 60–71	Both athletes continued to compete and experienced increases in strength and a decrease in pain during exercise and rest. S1 had a decrease in patellar tendon thickness, while S2 had an increase.	**JBI** 7/8 total yes
Hogsholt et al 2022 [34]	Adherence & adverse events Pain NRS & VISA-G EQ-5D VAS Oxford hip score HAGOS 30s chair stand test Stair ascending MVC hip abduction MVC hip extension MVC knee extension	96% adherence. one adverse event ↓ 50% (p < 0.001) & ↑ 18% (p = 0.046) ↑ 17% (0.009) ↑ 26% (p < 0.001) majority of subscales significant improved ↑ 33% (p < 0.001) ↑ 19% (p = 0.014) ↑ 26% (p < 0.001) ↑ 63% (p < 0.001) ↑ 20% (p = 0.031)	BRFT appears to be feasible for the treatment of gluteal tendinopathy with. high adherence, low drop-out rate, and improvements in pain levels, PROMs, and strength..	**JBI** 7/8 total yes
Kara et al. 2024 [29]	U/S Muscle thickness supraspinatus (SS). infraspinatus (IS). biceps brachii (BB). scapula retractor (SR)	SS. IS. SR was thicker after intervention. Groups differences were only seen for biceps with thicker muscle for BRFT.	BFRT resulted in a greater increase in biceps thickness and shoulder IR strength compared to the non-BFR group. However, neither exercise training showed superiority in terms of rotator cuff, scapula retractor, or deltoid muscle thicknesses, nor in improvements in shoulder external rotation strength and shoulder pain/function..	**D&B** 16/17
	Isokinetic torque Nm/kg – IR and ER 60°/s	**BFRT** ↑ 13 & 15% **LLRT** ↑2.4 & 9.7% (P = < 0.02 group p = 0.04 BRFT higher IR strength)		
	Isokinetic torque Nm/kg – IR and ER 180°/s	**BFRT** ↑ 15 &14% **LLRT ↑**8 & 23% (P = < 0.002 group p = NS)		
	VAS Pain activity	**BFRT** ↓85% **LLRT** ↓79% (P < 0.001. group p = NS)		
	Shoulder Pain and Disability index	**BFRT** ↓ 80.3% **LLRT** ↓ 63.1% (P < 0.001 group p = NS))		
Karanasios et al. 2022 [36]	Pain intensity Patient-rated tennis elbow evaluation Pain-free grip strength Global rating of change MVC elbow flexors MVC elbow extensors U/S elbow extensor tendon thickness	**BFRT** ↓ **LLRT-sham** ↓ p < 0.001 favor BRFT **BFRT** ↓ **LLRT-sham** ↓ p < 0.001 favor BRFT **BFRT** ↑ **LLRT-sham** ↑ p < 0.001 favor BRFT **BFRT** ↑ **LLRT-sham** ↑ p = 0.018 favor BRFT **BFRT** ↑ **LLRT-sham** ↑ p < 0.01 favor BRFT **BFRT** ↑ **LLRT-sham** ↑ NS **BFRT** ↓ **LLRT-sham** ↓ NS	BRFT produced significantly better results compared to LLRT-sham for all primary outcomes and at 6 and 12weeks. Patients in BRFT group had greater odds of reporting complete recovery. No significant differences for tendon thickness were observed for either time or group.	**D&B** 17/17
Karanasios et al. 2023 [35]	Pain intensity Patient-rated tennis elbow evaluation Pain-free grip strength Global rating of change Pressure pain threshold	↓ Week 3–42% Week 6 Pain-free ↓ Week 3–43%. Week 12 -.no symptoms ↑ Week 3 LSI 81%. Week 12 LSI 103% ↑ Week 12 complete recovery ↑ 21%	Adding wrist extensor exercise with BRFT seems like a promising approach to improve outcome in lateral elbow tendinopathy	**JBI** 7/8 total yes
Sata 2005 [51]	Circumference of thigh and calf Weighted MRI patella tendon VAS Pain Swelling	No evidence for muscle atrophy. Lower intensity of the signal. Decreased after 1week Decreased after 1week	Low-load BRFT was successfully performed, allowing the patient was quickly return to playing basketball. BRFT may be useful in rehabilitation of tendinopathy.	**JBI** 4/8 total yes
Skovlund 2020 [47]	Training compliance Pain last 7days & activities U/S tendon thickness U/S doppler vascularization MVC knee extension	98% ↓ 80% NS & ↓ 50% p < 0.05 Unchanged ↓ 31% doppler pixel area p < 0.05 ↑ 4% p < 0,05	Moderate to large pain improvements were reported, with no adverse events. The results show substantial clinical improvements over a 3week period and suggest that this could be an effective clinical rehabilitation tool in patients with chronic patellar tendinopathy. A reduction of vascularization was observed.	**JBI** 7/8 total yes
Wentzell 2018 [50]	Experienced strength improvement AROM - Elbow flexion AROM - Elbow extension AROM - Wrist pronation and supination Pain - Digit flexion and extension Pain - Elbow extension	↑ Week 9. 11. 12. 13. 14 ↑ Week 3. 5. Full AROM at week 11 ↑ Week 3. Full AROM at week 4 ↑ Week 3. 4 and full AROM at week 7* ↓ Week 1 Pain-free ↓ Week 2 Pain-free	BFRT enabled early loading and was successfully used as a part of a multimodal rehabilitation program after distal bicep brachii rupture and reconstructive surgery.	**JBI** 6/8 total yes
Yow et al. 2018 [49]	Isokinetic peak-torque - plantar flexion 60°/s	**S1** ↑ 4475% **S2** ↑ 68.80%	Both patients improved their ability to ambulate. experienced reductions in pain, and increased plantarflexion strength after the BFR intervention.	**JBI** 7/8 total yes
	Isokinetic peak-torque - plantar flexion 120°/s	**S1** ↑ 211.11% **S2** ↑ 78.74%		
	Power - plantar flexion 60°/s	**S1** ↑ 522.22% **S2** ↑ 55.82%		
	Power - plantar flexion 120°/s	**S1** ↑ 108.87% **S2** ↑ 47.06%		

All outcomes are measured after intervention and compared with pre-intervention measures. if nothing else is stated

**U/S =** Ultrasound. **JBI** = The Joanna Briggs Institute Critical Appraisal tools - Checklist for case reports (Supplementary) **D&B** = Modified downs and blacks - critical appraisal for clinical trials **BFRT =** Blood flow restriction training **LLRT =** Low load-resistance training **LIRT** = Low-intensity resistance training **HLRT =** High load-resistance training **CON =** Control group **S1 =** Subject 1 **S2 =** Subject 2 **1RM =** 1 repetition maximum **MVC =** Maximum voluntary contraction **CSA** = Cross-sectional area **RPE =** Rate of perceived exertion **AROM =** Active range of motion **VISA-*P*** **=** Victorian institute of sport assessment. patellar tendon (higher ratings = less impairment) **VAS =** Visual analogue scale **ADL =** Activities of daily life **P =** Probability value. **h =** hours **s =** second **MRI =** Magnetic resonance imaging **LSI** = limb symmetry index

### Critical appraisal

To assess methodological quality in randomized and non-randomized trials, a modified version of the Downs and Black scale was used [38]. The original Downs and Black scale has been validated and shown to be reliable when rating the quality of non-randomized and randomized controlled trials [39]. In our modified version, the maximum rating was 17 points. To assess the quality of case reports, the JBI Critical Appraisal Checklist for Case Reports was used [40]. Two of the authors (SÖ, ET) assessed each study independently using the appropriate assessment tool. After evaluation, ratings were compared and adjusted to achieve consensus. If there was disagreement, the third author was consulted to reach a consensus. The results of the methodological quality assessment are presented in the text and in Table 2 as well as expanded in tabular format (Supplementary Tables 1 and 2).

### Synthesis of results

In accordance with the PRISMA-ScR [37], the results are presented in a tabular synthesis with a narrative commentary. Study characteristics are presented in tabular format (Table 1), along with the main findings, conclusions and critical appraisal, which are also expanded upon (Table 2). Study characteristics, intervention, control conditions, and outcome measures are summarized in the text. Objective outcome measures, e.g., tendon thickness and peak torque, are converted and presented in percentages, pre- and post-intervention. Subjective measurements, e.g., patients’ perceived effort in activities of daily life and diagnosis-specific questionnaire, are also presented.

## Results

### Selection process

**Fig. 1:**
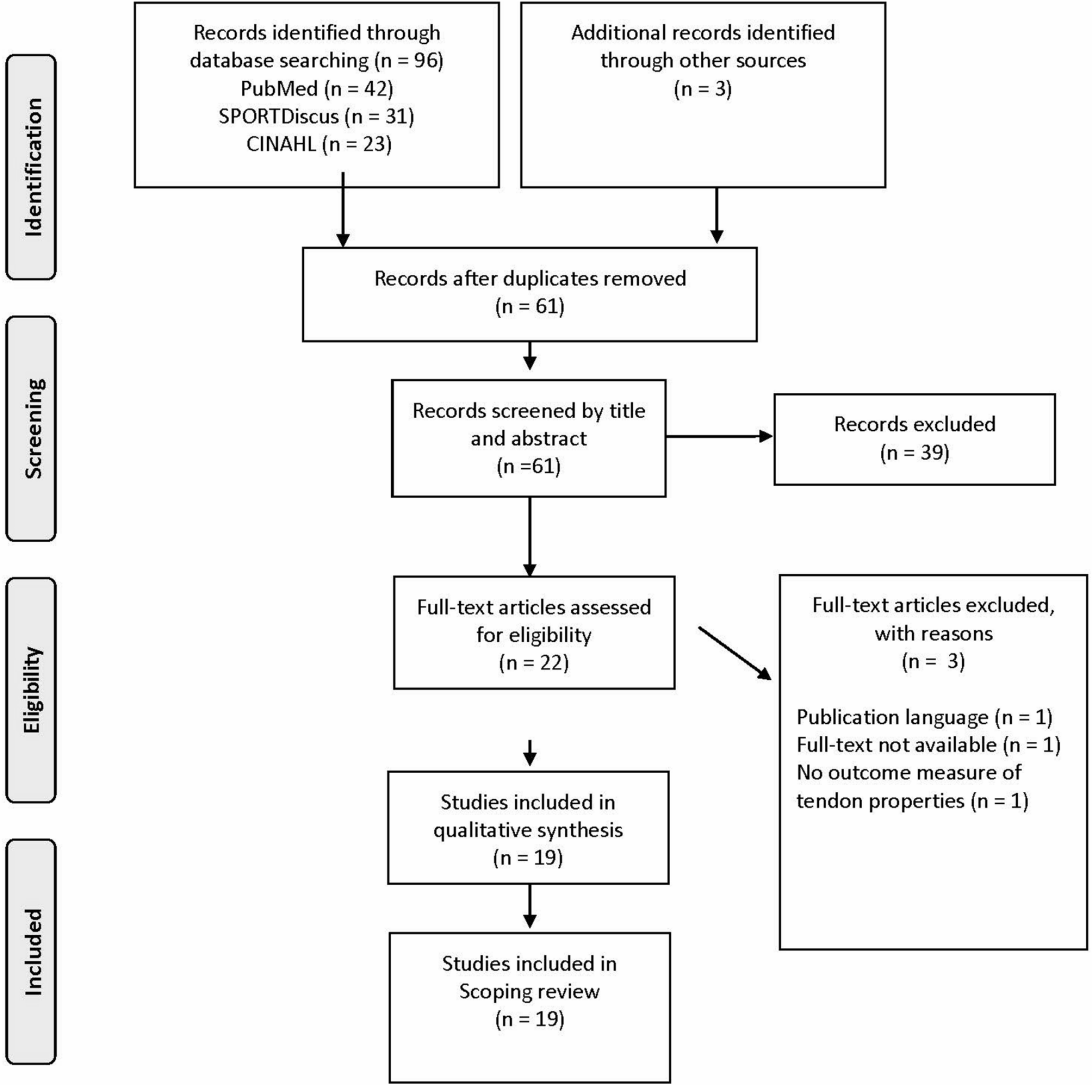
Flow chart of the inclusion process

The systematic search yielded a total of 61 articles that were included in the title and abstract screening. 39 articles did not meet our inclusion criteria and were therefore excluded. 22 articles were assessed for full-text eligibility and 19 were eligible for synthesis and inclusion in the present scoping review. Article screening and selection process followed a PRISMA flow chart (Fig. 1).

### Study characteristics

The included studies varied in design, population and investigated tendon (Table 1). Ten studies were randomized controlled trials (RCT), of which eight studies [31–33, 41–45] included healthy individuals and two studies [29, 36] included individuals with tendon-related disorders. Of the remaining studies, one was a clinical controlled trial [46], three were feasibility studies [30, 34, 47] and five were case reports [35, 48–51]. The sample size in the RCTs and the clinical trial [29, 31–33, 36, 41–46] ranged between 9 and 56 subjects, whereas case reports consisted of 1–2 persons [35, 48–51]. The studies involved a total of 351 healthy subjects [31–33, 41–46] and 122 individuals with tendon-related disorders, comprising 101 subjects with tendinopathy [29, 34–36, 47, 48, 51] and 21 subjects with tendon ruptures [30, 49, 50]. Six studies investigated the Achilles tendon [30, 32, 42, 44, 46, 49], six studies investigated the patella tendon [31, 41, 45, 47, 48, 51], one investigated hamstring [33], one investigated gluteal tendon [34], one investigated the biceps brachii distal tendon [50], two investigated tendons of the rotator cuff [29, 43], and two studies investigated lateral elbow extensors [35, 36].

### Description of the interventions and control conditions

Different intervention designs were used in the included studies (Table 1). BFRT at 20–35% 1RM was the most common load [29, 32, 33, 35, 36, 41–49, 51]. Training frequency ranged between 2 and 7 times per week with a duration of 3–15 weeks. Four sets per training session were most commonly used [29, 30, 32, 35, 36, 41–45, 48–50], while five studies performed two to three sets [31, 33, 34, 46, 51], and one study performed six sets [47].

Twelve studies had subjects doing repetitions of 30-15-15-15 [29, 30, 32, 35, 36, 42–45, 48–50], whereas seven studies used a different repetition scheme [31, 33, 34, 41, 46, 47, 51].

Furthermore, two studies [44, 46] investigated short-term effects, whereas the rest observed long-term effects. HLRT was applied as a comparison treatment (control condition) in six trials [32, 33, 41, 42, 44, 45]. Load varied between 70–85%/1RM and performed exercises were the same as in the BFRT group. LLRT was applied in five trials and was performed with the same conditions as the BFRT group [29, 36, 43, 44, 46]. One study performed whole-body training with different exercises in different groups [31]. In the majority of studies, the cuff was applied proximally to the tendon, while in three studies [29, 34, 43], the cuff was applied distally to the tendon (rotator cuff, gluteal tendinopathy). The applied cuff pressure varied between 30 and 80%, with 40–50% being the most commonly used range.

### Outcome measures in healthy individuals and tendon rehabilitation

For the studies on healthy individuals, different tendon property outcomes were measured: tendon thickness [31, 43, 44, 46], tendon CSA [32, 33, 41, 42] and tendon stiffness [41, 42, 45] were assessed with ultrasound (Table 2). Tendon stiffness was measured either through tendon elongation during maximum voluntary contraction [41] or through Young’s modulus [42].

Regarding tendon rehabilitation, four studies included specific tendon measurements, where tendon thickness was measured with ultrasound [36, 47, 48], ultrasound quantification of vascularization in tendon [47] and weighted MRI on tendon [51]. Other outcome measures after the tendon rehabilitation were: **pain levels** (VAS) during resting and loading [29, 30, 34, 35, 47–51] or pressure pain threshold [35], **strength measures** [29, 34–36, 47–49] or perceived strength [50]. **Performance** was measured during one-leg-hop, heel-rise, or chair-stand tests [30, 34, 48] and **self-reported diagnosis-specific function** was measured by different scores [29, 30, 34, 35, 48]. **Feasibility outcomes**, such as drop-out rate, adherence, and compliance, were also measured in three studies [30, 34, 47].

### Result synthesis

In the nine studies on healthy individuals, the effects of BFRT show contradictory results. Four studies showed tendon adaptations during BFRT conditions on Achilles-, supraspinatus-, and patella tendons [32, 42, 43, 45] while three studies on quadriceps-, patella, and hamstrings tendons did not [31, 33, 41]. However, changes in outcome measures did not differ significantly from HLRT conditions [32, 42, 45] or LLRT conditions [43], albeit with one exception in which one study showed a larger increase of MRI CSA patellar tendon compared to HLRT [45]. Four studies reported significant increases in tendon thickness for Achilles-, supraspinatus-, and patella tendon [32, 42, 43, 45], and one study demonstrated a significant increase in Achilles tendon stiffness [42]. Kubo et al., the control condition reported a significant increase for patella tendon-aponeurosis stiffness and tendon stiffness [41]. Two studies measured short-term adaptations and reported significant decreases in Achilles tendon thickness acutely as well as 24 h post-intervention [44, 46].

Regarding tendon rehabilitation, the four studies that included specific tendon property measurements showed contradictory results. Two studies—one RCT (*n* = 46) on elbow extensor tendon and one feasibility study on patellar tendon (*n* = 7)—reported no significant differences in tendon thickness [36, 47], while one case study (*n* = 2) reported an increase in patellar tendon thickness for one subject, but a decrease for the other subject [48]. In addition, one case study on patellar tendinopathy (*n* = 1) reported lower signal intensity on weighted tendon MRI [51], and one feasibility study on patellar tendinopathy (*n* = 7) reported significantly decreased Doppler pixel area when quantifying vascularization in the patellar tendon [47].

All studies reported a decrease in pain [29, 30, 34–36, 47–51] and several patients became pain-free after BRFT. BRFT also increased strength [29, 34–36, 47–50] and performance [30, 34, 48], and improved self-reported diagnosis-specific function [29, 30, 34, 35, 48].

Two included studies were RCTs. In one of the RCTs on rotator cuff tendinopathy, the improvements were equal for BRFT and LLRT, except for better improvement of internal rotation strength for BRFT [29]. The other RCT on lateral elbow tendinopathy presented significantly better results for BRFT compared to LLRT-sham for all primary outcomes (pain intensity, self-reported function, strength) [36].

### Critical appraisal

Eleven articles were reviewed with the modified Downs and Black scale with a mean score of 79%, ranging between 53 and 100% (Table 2 and Supplementary Table 1). Questions concerning overall study design and reporting (Q1-7, 10) received an average score of 92%, ranging between 63 and 100%. Questions regarding internal validity averaged 82%, ranging between 67 and 100%. Four studies [29, 31, 36, 42] demonstrated sufficient power (Q27). Eight case reports were reviewed with the JBI Critical Appraisal Checklist for Case Reports and scored between 4 and 7 of 8 “YES”, with six articles with a score of 7/9 “YES” (Table 2 and Supplementary Table 2). The case reports were thus deemed to be of high quality.

## Discussion

BFRT is gaining traction in tendon research and several studies have recently been published using the method in both tendon rehabilitation settings and in healthy populations. The present scoping review summarizes 19 studies, of which nine studies investigate the effects of BFRT in healthy subjects [31–33, 41–46] and ten studies use BFRT in a tendon rehabilitation setting [29, 30, 34–36, 47–51]. Included studies were of different designs, sampling various populations and pathologies and using BFRT both distally and proximally to the target tendon.

### Blood flow restriction training and tendon adaptation

The results in our review were contradictory concerning the effect of BFRT on tendon adaptation, where both an increase [32, 42, 43, 45], and no significant changes [31, 33, 41] were demonstrated in tendon stiffness or tendon CSA. An early study investigating the impact of BFRT on tendon adaptation was Kubo et al. 2006, who compared the effect of BFRT versus HLRT on the patellar tendon after a 14-week intervention [41]. They showed an increase in patellar tendon stiffness only in the HLRT group, while no change in tendon CSA was noted in either group. This result was in line with earlier research demonstrating the need for high loads to achieve tendon adaptations [1]. However, in a study by Centner et al. 2019 [42], significant Achilles tendon stiffness and tendon CSA increases were demonstrated during both HLRT and LLRT conditions, challenging previous notions. Further, using the same study design, results were replicated in the patellar tendon [45] and later demonstrated that regional hypertrophy in the Achilles tendon is similar during BFRT and HLRT conditions. Centner et al. suggest that these discrepancies might be attributable to differences in study design, such as inadequate load progression, or inadequate sensitivity in load measurement techniques. Three RCTs on the semitendinosus, quadriceps and supraspinatus tendons demonstrate no significant group or time effect on tendon thickness. However, these studies were of short duration, 6–9 weeks, and tendon adaptation seems to occur at a slower rate [1], and the three studies by Centner et al. showing promising results on BFRT’s capability to achieve tendon adaptation had an intervention length of 14 weeks [32, 42, 45].

Chulvi-Medrano et al. [46] and Picon-Martinez et al. [44] investigated short-term effects of BFRT on Achilles tendon thickness. Both studies measured a decrease in tendon thickness acutely and at 24 h post-training. Earlier studies have demonstrated a transitory decrease in tendon thickness post-HLRT [52–54]. This has been attributed to a shift of fluid from the tendon matrix to the peritendinous area [55] and has been hypothesized to be a beneficial marker of tendon adaptation [52, 54, 56]. Earlier studies have shown that BFRT requires less total workload to achieve fatigue than LLRT [57]. At 30% of 1RM, LLRT achieves more than twice the number of repetitions over four sets than BFRT [58]. Higher loads have also been shown to acutely decrease tendon thickness [52, 58, 59]. The acute reduction in tendon thickness has been regarded as a positive adaptation [52, 54] but long-term effects and importance in tendon rehabilitation are unknown. Meta-analyses investigating correlations between structural tendon adaptation and clinical outcomes in tendinopathy show contradictory results [60–62] and need to be further investigated. The mechanisms behind tendon adaptations observed during BFRT are unclear. However, it is proposed that the state of reduced blood flow and ischemic environment initiates an effect on collagen metabolism and tendon remodeling [63]. Additionally, the systemic effects of BFRT should be considered as earlier research has shown effects on neurological function, metabolic, endocrine and skeletal systems [24, 64, 65]. For example, LL-BFR has been observed to improve bone markers [64], an effect that might extend to the tendinous system.

### Blood flow restriction training in tendon rehabilitation

Studies on BFRT on pathological tendons are of varied study design, tendon and methodological quality. To summarize the ten included studies, RCTs on lateral elbow tendinopathy and rotator cuff tendinopathy demonstrated superior [36] and comparable [29] results in strength and functional outcomes in comparison to LLRT. Three feasibility studies demonstrated high adherence to BFRT after Achilles tendon rupture [30], in gluteal tendinopathy [34] and in patellar tendinopathy [47]. Additionally, five case reports using BFRT demonstrated improvements in functional outcomes and pain [35, 48–51]. However, one study reported serious adverse events where two Achilles re-ruptures and one deep vein thrombosis occurred [30].

One of the RCTs [36] compared BFRT to LLRT (sham BFRT) in lateral elbow tendinopathy, and demonstrated significantly better results both in patient-reported outcomes such as pain and function and in strength measures. Results on a patient-reported tennis elbow evaluation questionnaire showed clinically significant results for disability at both 6 and 12 weeks. Regarding lateral elbow tendinopathy, reviews demonstrate superior effects of exercise interventions in comparison to passive interventions. However, evidence is of low quality and results do not reach clinical significance [66, 67]. With the clinically significant results of Karaniosos et al. [35, 36], this study might be used as a basis for BFRT rehabilitation in the rehabilitation of lateral elbow tendinopathy.

Studies have investigated muscles and tendons using both proximal and distal BFRT applications even though, a proximal application is more common. Kara et al. investigated the effects of BFRT on rotator cuff tendinopathy using a distal cuff application and found superiority for BFRT on bicep brachii thickness and internal rotation strength, while other outcomes for rotator cuff and scapular strength, shoulder function and hypertrophy showed only significant time effects. The authors hypothesize that the increases in internal rotation strength might be attributable to effects on larger muscle groups such as pectoralis major. Further, a RCT study by Brumitt et al. on healthy individuals, demonstrated significant time effects of BFRT on rotator cuff strength and supraspinatus tendon thickness [43]. Furthermore, a feasibility study by Hogsholt et al. investigated the use of BFRT on patients with gluteal tendinopathy using an application distal to the target tendon. The study demonstrated decreased pain and increased performance in functional outcomes [34]. Although these studies are of varied quality and should be interpreted with caution, they point towards benefits of distal application. These results are in line with a systematic review investigating proximal and distal applications of BFRT, which found hypertrophy and strength increases during both conditions when comparing BFRT to HLRT [68]. Strength and hypertrophy increase after proximal BFRT-application have been demonstrated both in the upper- and lower extremities [69, 70]. However, tendon adaptation to proximal BFRT remains unclear.

In terms of feasibility, three case series noted training adherence between 88 and 96% in a total sample of 41 individuals, both male and female, athletic and sedentary. Moreover, acceptability to intervention was recorded at 92%, highlighting the acceptability of BFRT [30, 34, 47]. Across case and feasibility studies, improvements in functional capacity and strength and decreases in pain were reported [30, 34, 35, 47–51]. Studies on tendon ruptures using BFRT showed enabling of early loading, reduction of pain, and improved function, both self-reported and measured through functional tests such as strength [30, 49, 50].

Case and feasibility studies on tendinopathy in different populations demonstrated good implementation of BFRT in rehabilitation [34, 35, 47]. The case reports demonstrate how a BFRT intervention may enable high relative intensity and increased strength and function. Tendon-related load pain is common in tendinopathy, and BFRT might be most useful in the early phase of tendon rehabilitation to allow loading with high relative intensity and low absolute load, thus decreasing load-related pain. BFRT also shows increased rates of strength and muscle mass adaptations in comparison to LLRT [24, 25].

In tendinopathy, tendon structure and function are affected. A systematic review by Obst et al. [71] demonstrated conflicting evidence regarding tendon mechanical and material adaptations in the Achilles and patellar tendons. Both conditions led to an increase in tendon CSA. For Achilles tendinopathy, the included studies consistently showed a decrease in tendon stiffness and an increase in tendon strain for the same amount of force applied. In contrast, results regarding patellar tendinopathy are conflicting and both increased and decreased tendon stiffness have been demonstrated [71]. In our review, four tendinopathy studies measured specific tendon measurements. Regarding tendon thickness, an RCT on lateral elbow tendinopathy [36] found non-significant changes during both BFRT and LLRT-sham conditions, while BFRT demonstrated superior functional-, strength- and pain-related outcomes. However, this intervention lasted only 6 weeks and might thus be insufficient to induce changes in tendon thickness [1]. Interestingly, a case series on seven individuals with patellar tendinopathy noted no changes in tendon thickness but demonstrated a significant decrease in tendon vascularization after only 3 weeks of BFRT with simultaneous moderate-to-large pain reduction [47]. An earlier systematic review found moderate evidence for a correlation between tendon neovascularization and clinical outcomes in tendinopathy [62], and interventions that achieve a decrease in vascularization warrant further investigation. Due to most studies on BFRT and tendon rehabilitation being non-controlled studies, there is insufficient evidence to draw conclusions on effects. However, the recency of published studies indicates the method is being used to a greater degree in tendon rehabilitation.

Concerns about the safety of BFRT have been raised in earlier research, but the literature is scarce and the generalizability between populations is limited [72–74]. One narrative is that BFRT might induce abnormal cardiovascular responses in cardiac disease patients [74], thus questioning the safety of BFRT. The present review included 122 subjects in a tendon rehabilitation setting and three serious adverse events were recorded. In one feasibility study, two Achilles tendons re-ruptured and one deep vein thrombosis (DVT) occurred [30]. This highlights the need for further investigation of the safety of BFRT in certain populations. Nevertheless, preliminary evidence points towards BFRT being a tolerable and feasible training method in tendon rehabilitation.

### Recommendations for future research

To better understand BFRT’s effect on tendons, future research should investigate the mechanism behind tendon adaptation during BFRT. Interventional studies comparing BFRT to LLRT could include training to volitional failure. The application of BFRT lowers the number of repetitions needed to reach failure in comparison to LLRT [57] and the amount of workload to reach failure is therefore higher in LLRT [58]. In addition, LLRT executed to failure increases strength, although to a lesser degree than BFRT [24, 25]. Progression in training load should also be a feature of future studies. To draw accurate conclusions, studies need to address specific tendons and pathologies. This scoping review found adaptations in the Achilles and patella tendons in a healthy population, and improvements in functional outcomes and pain in subjects with lateral elbow tendinopathy. Thus, demonstrating beneficial effects on both high-force absorbing tendons and positional, force-transferring tendons. However, differences in tendon load, function and pathology should be taken into consideration as these differences might affect training responses. To further clarify the effects of BFRT, application, width, and pressure need to be standardized. In the present review, the applied cuff pressure varied between 30 and 80%, with 40–50% being the most commonly used range. An arterial occlusion pressure of 40–80% has been shown to produce similar strength adaptations [12, 21]. Further, the method for measuring and determine the arterial occlusion pressure needs to be standardized. Study intervention length should be considered when designing further tendon rehabilitation studies. RCTs are also needed to further detail the effects of BFRT in comparison to other loading programmes such as heavy slow resistance training or eccentric training.

### Methodological considerations

This scoping review was conducted according to the PRISMA-ScR. To ensure that the literature search was comprehensive and systematically designed, a medical librarian was consulted. A strength of the scoping review method is that it enables a multitude of different data to be compiled. This was of great importance due to the heterogeneity of the literature and ensured a comprehensive analysis of the available literature. Inclusion in the present scoping review was limited to peer-reviewed research. Therefore, grey research and published research without peer review were excluded. This was done to achieve higher article quality but may have resulted in exclusion of relevant articles.

## Conclusions

Nine studies have investigated BFRT’s effect on tendon adaptation in healthy subjects and ten studies have been published on BFRT in tendon rehabilitation. Preliminary evidence points towards BFRT as a training modality capable of achieving tendon adaptation in healthy subjects and increasing tendon function after rehabilitation. BFRT may be a viable option to incorporate into training regimes aimed at inducing tendon adaptation in both healthy and pathological tendons. Since the majority of the literature concerns non-controlled studies, generalizability is low, and conclusions should be made with caution. It is recommended that future studies investigating the effect of BFRT on pathological tendons compare BFRT to either HLRT or LLRT for a duration of at least 12 weeks while equating training effort and using similar progression between groups. Further research is needed to comprehensively understand BFRT and its use in tendon rehabilitation.

## Electronic supplementary material

Below is the link to the electronic supplementary material.

Supplementary Material 1

## Data Availability

No datasets were generated or analysed during the current study.

## Abbreviations

1RM: Repetition maximum
BFRT: Blood flow restriction training
HLRT: High load resistance training
LLRT: Load resistance training
