# Supplementary material for: The impact of blood flow restriction training on tendon adaptation and tendon rehabilitation – a scoping review
Source: BMC Musculoskelet Disord. 2025 May 22;26:503. doi: 10.1186/s12891-025-08734-5 (PMC12096532; doi:10.1186/s12891-025-08734-5)
Supplement: Supplementary file 1 — Supplementary Material 1 [file 12891_2025_8734_MOESM1_ESM.docx]

Supplementary data

Supplementary materials

**Search strategy**

**CINAHL** Filter; abstract

(“blood flow restriction*” OR “occlusion training” OR kaatsu OR “occlusion exercise*” OR “vascular occlusion” OR “BFR”) AND (Tendon OR tendinopathy* OR “structural adaption*”)

**Pubmed** Filter; abstract

(“blood flow restriction*”[tiab] OR “occlusion training”[tiab] OR kaatsu[tiab] OR “occlusion exercise*”[tiab] OR “vascular occlusion”[tiab]  OR “BFR”[tiab])  AND (Tendon[tiab] OR tendinopathy*[tiab] OR “structural adaption*”[tiab])

**SportDiscus**

(“blood flow restriction*” OR “occlusion training” OR kaatsu OR “occlusion exercise*” OR “vascular occlusion” OR “BFR”) AND (Tendon OR tendinopathy* OR “structural adaption*”)

## Supplementary Table 1. Critical appraisal for clinical and randomized controlled trials - Modified Downs and Black

|  | **Q1** | **Q2** | **Q3** | **Q5** | **Q6** | **Q7** | **Q10** | **Q15** | **Q16** | **Q18** | **Q20** | **Q21** | **Q22** | **Q25** | **Q27** | **Total score** |  |
| --- | --- | --- | --- | --- | --- | --- | --- | --- | --- | --- | --- | --- | --- | --- | --- | --- | --- |
| **First Author** | **Reporting** | | |  |  |  |  | **Internal validity** | | | | |  |  | **Power** | |  |
| Brumitt 2020 | Y | Y | Y | P | Y | Y | Y | Y | Y | Y | Y | Y | UD | Y | UD | 14/17 | 82% |
| Centner 2019 | Y | Y | Y | Y | Y | Y | Y | Y | Y | Y | A | Y | UD | Y | Y | 15/17 | 88% |
| Center 2022 | Y | Y | Y | Y | Y | Y | Y | Y | Y | Y | A | Y | UD | N | N | 13/17 | 76% |
| Centner 2023 | Y | Y | Y | Y | Y | Y | N | Y | Y | Y | A | Y | UD | N | N | 13/17 | 76% |
| Chulvi-Medrano 2020 | Y | Y | Y | P | N | Y | N | UD | Y | Y | A | Y | UD | N | UD | 9/17 | 53% |
| Cintineo 2024 | Y | Y | Y | Y | Y | Y | Y | UD | Y | Y | A | Y | UD | N | Y | 13/17 | 76% |
| Frouin 2024 | Y | Y | Y | Y | Y | Y | Y | Y | Y | Y | A | Y | UD | UD | UD | 13/17 | 76% |
| Kara et al 2024 | Y | Y | Y | Y | Y | Y | Y | Y | Y | Y | A | Y | Y | Y | Y | 16/17 | 94% |
| Karanasios2022 | Y | Y | Y | Y | Y | Y | Y | Y | Y | Y | Y | Y | Y | Y | Y | 17/17 | 100% |
| Kubo 2006 | Y | Y | UD | Y | Y | Y | Y | N | Y | Y | A | Y | UD | Y | UD | 12/17 | 71% |
| Picón-Martínez 2021 | Y | Y | Y | Y | Y | Y | Y | UD | Y | Y | Y | Y | UD | UD | UD | 13/17 | 76% |
| Y=Yes N=No UD=Unable to determine A=Accuracy not reported but method clearly described. P=Partially  2 points are given for Yes (Y) regarding questions 5 and 20, 1 point is given for Yes (Y), Accuracy (A) and Partially (P) regarding the remainder of questions, No points are given for No (N) nor for Unable to determine (UD). Q1 – Aim/hypothesis, Q2 – main outcome description, Q3 - subjects characteristics, Q5 – Distribution of confunders, Q6 – Main findings, Q7 – estimates of the random variability, Q10 – actual probability, Q15 – Blinding, Q16 – if data dredging was made clear, Q20 – description of valid and reliable measures, Q21 – population recruited from same population, Q22 – recruited over same time period, Q25 – adjustments for confounders, Q27 – sufficient power. | | | | | | | | | | | | | | | | | |

Supplementary Table 2. Critical appraisal for case rapports - JBI

| **First Author** | **Q1** | **Q2** | **Q3** | **Q4** | **Q5** | **Q6** | **Q7** | **Q8** | **Total score** | |
| --- | --- | --- | --- | --- | --- | --- | --- | --- | --- | --- |
| Bentzen 2024 | Y | N | Y | Y | Y | Y | Y | Y | 7/8 | 88% |
| Cuddeford 2020 | Y | Y | Y | Y | Y | Y | N | Y | 7/8 | 88% |
| Hogsholt 2022 | Y | N | Y | Y | Y | Y | Y | Y | 7/8 | 88% |
| Karanasios2023 | Y | Y | Y | Y | N | Y | Y | Y | 7/8 | 88% |
| Sata 2005 | Y | N | N | Y | N | N | Y | Y | 5/8 | 50% |
| Skovlund 2020 | Y | N | Y | Y | Y | Y | Y | Y | 7/8 | 88% |
| Wentzell 2018 | Y | Y | Y | Y | Y | UC | N | Y | 6/8 | 75% |
| Yow 2018 | Y | Y | Y | Y | Y | Y | N | Y | 7/8 | 88% |
| Y=Yes, N=no, UC=unclear. Q1-Demograhic characteristics, Q2 - Patient´s history and timeline, Q3 - Clinical condition (pre),  Q4 - Diagnostic test or assessment methods, Q5 – Intervention or treatment description, Q6 – Clinical condition (post), Q7 – Adverse events, Q8 – Takeaway lessons | | | | | | | | | | |
